# Influence of different layout of rigid and flexible structures on methane explosion in oil and gas gathering and transportation station

**DOI:** 10.1371/journal.pone.0342568

**Published:** 2026-02-20

**Authors:** Weifeng Duan, Shitong Liu, Kan Du, Yulong Duan, Huaixu Liu

**Affiliations:** 1 Natural Gas Processing Plant, Zhongyuan Oilfield of China Petroleum & Chemical Corporation Henan, Henan, China; 2 College of safety science and Engineering, Chongqing University of Science and Technology Chongqing, China; Southwest Petroleum University, CHINA

## Abstract

Oil and gas gathering and transportation station is the core hub of oil and gas production, processing and transportation. Its safe operation is directly related to the stability of energy supply and the safety of personnel and property. To solve the problems of high explosion risk, limited overpressure attenuation and facilities vulnerable to explosion impact damage caused by rigid facilities in the oil and gas gathering and transportation station, based on the small-scale explosion experimental platform, this paper studies the space protection technology of flexible facilities, and discusses the gas explosion-proof effect of different sizes of flexible facilities in front of rigid facilities. The experimental results show that in most cases, the flame front velocity and flame explosion overpressure are positively correlated with the blockage rate of the flexible structure; When the blockage rate of flexible structure is less than or equal to the blockage rate of rigid structure, the flame velocity and explosion overpressure decrease with the decrease of the spacing, and the explosion protection effect is better; When the blockage rate of flexible structure is greater than the blockage rate of rigid structure, the smaller spacing may aggravate the explosion. The research results help to clarify the influence mechanism of flexible protective facilities on natural gas explosion pressure and flame characteristics, reveal the protection mechanism of flexible protective facilities on rigid facilities, and provide theoretical basis and scientific guidance for the protection layout design of rigid facilities and natural gas explosion protection technology in oil and gas gathering and transportation station.

## Introduction

**Fig 1:**
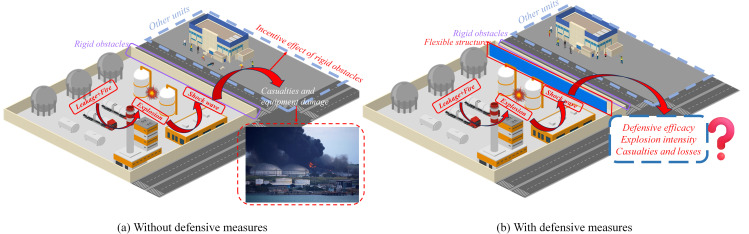
Oil and gas gathering and transmission station diagram.

With the rapid development of the world economy, the energy reserve strategy and production demand of the petrochemical industry are increasing, and the scale of oil and gas gathering and transportation stations is also expanding, and continues to develop in the direction of large-scale and intensive [1]. As the key link of oil and gas production, oil and gas gathering and transportation stations have typical flammable and explosive characteristics: on the one hand, dense pressure pipelines and storage and transportation equipment constitute a complex spatial structure; On the other hand, the explosion risk of oil and gas itself has significantly increased the accident risk [2]. The complexity of these densely arranged equipment, coupled with the surrounding wall as rigid obstacles, will intensify the explosion intensity and have a severe impact on the environment outside the station Existing studies have shown that rigid obstacles in such environments can significantly affect key parameters such as flame front velocity and explosion overpressure by enhancing turbulence and reflected pressure, thereby significantly increasing the risk of explosion [3,4]. Consequently, examining the influence of obstacles on the explosion characteristics of methane/air-premixed gas in explosive environments can furnish a scientific foundation for investigating the propagation dynamics of gas explosions in oil and gas gathering and transportation stations. As shown in Fig 1(a), it represents the situation when an explosion occurs in an oil and gas gathering and transportation station without using flexible facilities. The facilities and equipment in the station have been used for a long time and have not been repaired and maintained in a timely manner, resulting in leakage. When reaching a certain concentration and encountering open flames, an explosion will occur. The peripheral wall of the station, as a rigid structure, will enhance the explosion intensity during the explosion process, thereby expanding the casualties and property losses caused by other units.

Currently, the rigid and flexible structures in the explosion environment affects the flame propagation characteristics of the study is mainly focused on the flame state [5], the flame front propagation speed [6] and the flame explosion overpressure [7]. Chapman [8] found that the presence of obstacles in the flame pipe significantly affects the flame speed; the flame front speed can be increased by up to 70 times. Subsequent scholars for the presence of obstacles to the flame combustion and explosion characteristics of the research mainly focused on the shape of the obstacles, blockage rate, and the number and location in the pipeline. Wang [9] et al., using a horizontal long straight explosion experimental pipeline, studied a variety of shapes of obstacles on the flame propagation speed and gas explosion overpressure. Yu [10] et al., through the independent design and construction of a small experimental platform, researched methane flame propagation characteristics under multiple blockage rates. The results showed that the gradient of blockage rate has a large effect on the instantaneous flame speed, while the average speed does not have much effect. Duan [11] et al. designed three types of obstacle layouts, i.e., symmetrical, ipsilateral, and staggered. The results showed that the explosion hazard was greater when the obstacles were symmetrically distributed. Obstacles are mainly considered rigid facilities such as equipment and pipelines that are common in many facilities, however, in an explosion environment, there are also flexible facilities such as shrubs and vegetation, etc. Bakke [12] et al. used FLACS for modeling and found that trees and bushes, etc., accelerated the flames continuously, and Leal et al. [13] showed that the height of the trees and the spacing of the trees enhanced the explosion intensity by numerical simulation. Gao [14] et al. developed a numerical simulation model using CFD and found that the flexible piping system could cause many wrinkles in the flame shape. Yu [15] et al. found that the flexible material could absorb the reflected wave more efficiently than the rigid material and absorb the pressure oscillations to reduce the flame pressure peak. Duan [16] et al. investigated the combustion and explosion characteristics of hydrogen/methane premixed gases in a confined space under the coexistence of flexible and rigid obstacles and found that the explosion hazard was significantly increased under the preset conditions of rigid obstacles compared with flexible structures. This provides new ideas and a theoretical basis for studying rigid/flexible structures in the explosion of methane/air-premixed gases.

Most of the previous studies by scholars mainly focused on the purely rigid structures, and a few purely flexible structures to the flame propagation characteristics, while the coexistence of the study’s rigid and flexible flame propagation characteristics is less. The research on the coexistence of rigid and flexible explosive environment by a few scholars also focuses on the shape of flexible structures, blockage rate, etc. On this basis, this study focuses on the coexistence of rigid and flexible structures in oil and gas gathering and transmission stations. Considering the complexity of oil and gas gathering and transmission stations, the study focuses on the blockage rate of flexible structures and the changes in the spacing between rigid and flexible structures. The study explores the influence of flexible structures on the propagation of explosion flames under various working conditions, investigates the protective effect of flexible structures, and obtains a more optimal layout of rigid and flexible structures in oil and gas gathering and transmission stations to reduce explosion hazards. The study conducts experimental research in combination with the actual scenarios of oil and gas gathering and transmission stations. The research results provide a theoretical basis for the layout design of rigid and flexible structures around the station, guiding the optimization of the layout of oil and gas stations in combination with the dimensions of rigid structures around the station, and offering a more scientific approach to reduce the risk of methane explosion.

## Experimental setup and methods

**Fig 2:**
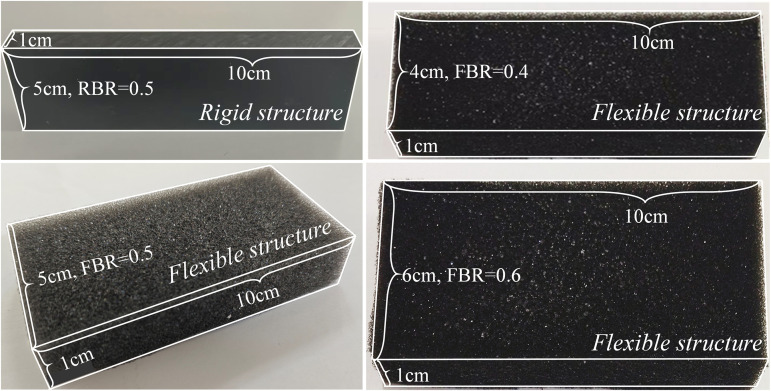
Rigid and flexible structure figure.

In this study, different layouts were used, specifically referring to the differences in size parameters of the rigid and flexible structures inside the pipeline, including the height of the flexible structure and the spacing between the rigid and flexible structures (corresponding to different operating conditions in Table 1). Other experimental conditions (such as methane concentration, initial pressure, etc.) were kept consistent to explore the effect of the size of the rigid and flexible structures inside the pipeline on methane explosion characteristics as a single variable. Based on previous research to further improve the flexible structure blockage rate and rigid and flexible structure spacing, this paper is designed as shown in Table 1 of the experimental conditions. In this paper, transparent pipes are used to simulate the explosion environment in Oil and gas gathering and transportation station, rigid structures simulate objects that are not easy to deform in the explosion scenario, and flexible structures simulate objects that are easy to deform in the explosion scenario. A carbon fiber board replaces the rigid structure, and the flexible structure is replaced by polyurethane foam wool (Fig 2). According to Masri [17], square obstacles have the greatest flame propagation velocity for methane explosions, so a square shape is chosen for rigid and flexible structures here.

**Table 1:** Design of experimental conditions.

Working condition	Rigid and flexible structure thickness/cm	Rigid structure blockage rate (RBR)	Flexible structure blockage rate (*FBR*)	Structure distance(*L*)/cm	Configuration purpose
C1	1 cm	0.5	–	–	Control group
C2			0.4	2 cm	FBR < RBR
C3				4 cm	
C4				6 cm	
C5			0.5	2 cm	FBR = RBR
C6				4 cm	
C7				6 cm	
C8			0.6	2 cm	FBR > RBR
C9				4 cm	
C10				6 cm	

The height of the rigid structure studied in this paper is 5 cm; to study the protective effect of the flexible structure on the rigid structure in the explosion environment, the flexible structure is placed in front of the rigid structure. In order to explore the protective effect of the flexible structure in the three cases of FBR < RBR, FBR = FBR, FBR > RBR, the height of the flexible structure is set to 4 cm, 5 cm, and 6 cm. According to the research of Yu [15], the rigid structure is placed 40 cm away from the ignition end in this paper; to explore the effect of the rigid-flexible structure spacing on the explosion characteristics of methane by varying the spacing of the rigid-flexible structure, the spacing (*L*) is selected as 2 cm, 4 cm, 6 cm. The detailed working conditions of the experiment are shown in Table 1. For the accuracy of the experimental data, each group of experiments is repeated 3 ~ 4 times.

This experiment involves the change of two variables: the blockage rate of the flexible structure and the spacing between the rigid and flexible structures. The blockage rate of the rigid-flexible structure at rest is calculated as follows:


$$\begin{array}{c} BR=\frac{h}{H} \end{array}$$ (1)

In eq. (1), h is the height of the rigid-flexible structure at rest, and H is the height of the transparent pipe.

So, in this study, the rigid structure blockage rate is fixed, i.e., RBR = 0.5, and the flexible structure blockage rates are 0.4, 0.5, and 0.6, respectively.

**Fig 3:**
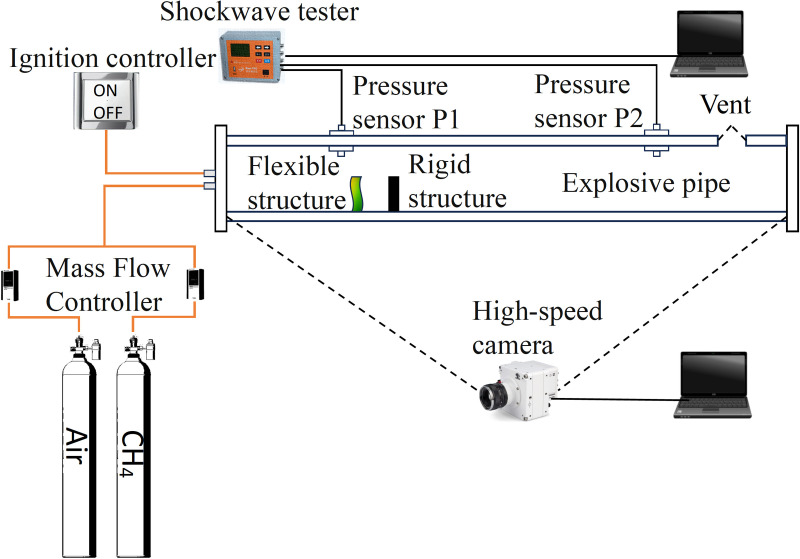
Experiment setup.

To restore the explosion scene in Oil and gas gathering and transportation station as much as possible, this paper designs a simplified experimental setup, as shown in Fig 3, including an experimental pipeline, ignition system, gas distribution system, high-speed camera system, and pressure acquisition system.

The experimental pipeline consists of 100 mm × 100 mm × 1000 mm transparent square pipe and square structure; square pipe can withstand the pressure of 2Mpa, and pipe wall thickness of 20 mm. The front and rear ends of the pipeline are sealed with 10 mm thick steel plates and sealant strips. The front steel plate is pre-installed with an ignition device and air inlet, and the rear steel plate is reserved with an exhaust outlet. A 30 mm diameter explosion vent is opened at the end above the pipeline. In all experiments, the vent is covered and sealed with PVC film [15].

The gas distribution system consists of air cylinders, methane cylinders and two mass flow meters. Methane purity is 99.99%, and concentration is 9.5%. Ambient air is used for the air, and the mass flow meters are high-sensitivity mass flow controllers from Alicat (range 0–5 L/min, meter error ±0.4% of reading). In order to avoid the impact of uneven gas mixing on the experimental results, the four volume method was used in the experiment, and a gas volume equivalent to four times the volume of the pipeline was introduced into the pipeline to ensure that the exhaust gas in the pipeline was discharged and the air and methane were mixed evenly [18]. After the ventilation was completed, the pre mixed gas in the pipeline was also left to stand for about 30 seconds for better mixing effect.

The ignition system comprises a homemade ignition head, a high-frequency pulse igniter, an ignition controller and an ignition power supply. The power supply’s input voltage is 6 V. At the ignition point, two platinum wires with a diameter of 0.1 mm generate the high-temperature spark.

The high-speed camera system consists of a PhantomV710L high-speed camera with a camera pixel setting of 1280 x 240 and a shooting speed of 2000 fps.

The pressure acquisition system consists of two pressure sensors. Pressure sensor 1 (hereinafter referred to as “*P1*”) is installed at a distance of 17.5 cm from the ignition head, and pressure sensor 2 (hereinafter referred to as “*P2*”) is installed at a distance of 77.5 cm from the ignition head to collect the signal of the pressure change at both ends of the pipeline. The pressure sensor range is 0 ~ 690 kPa, and the linearity error is less than 1%.

## Analysis of experimental results

### Flame propagation characteristics in placing flexible structure in front

**Fig 4:**
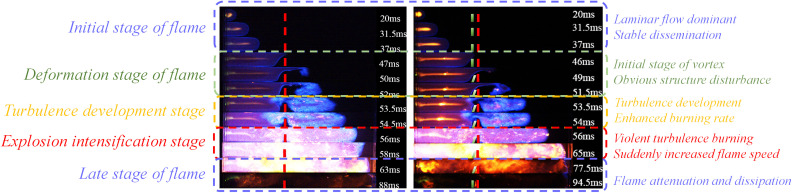
Flame stage division and characteristics.

**Fig 5:**
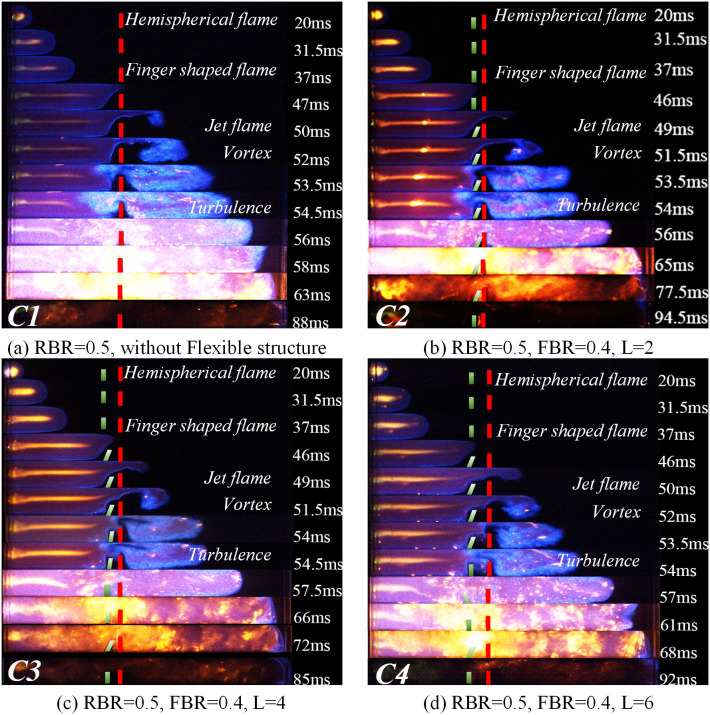
Flame Propagation Law at FBR = 0.4.

**Fig 6:**
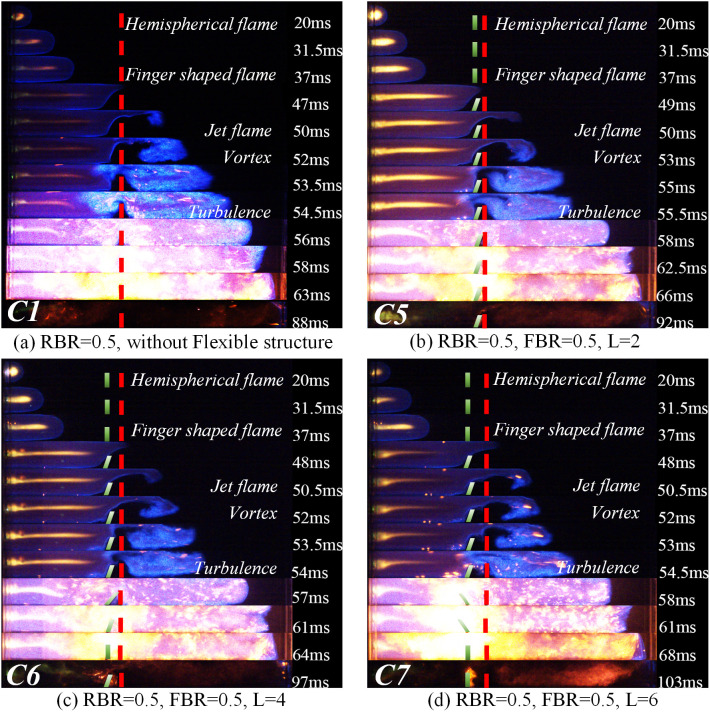
Flame Propagation Law at FBR = 0.5.

**Fig 7:**
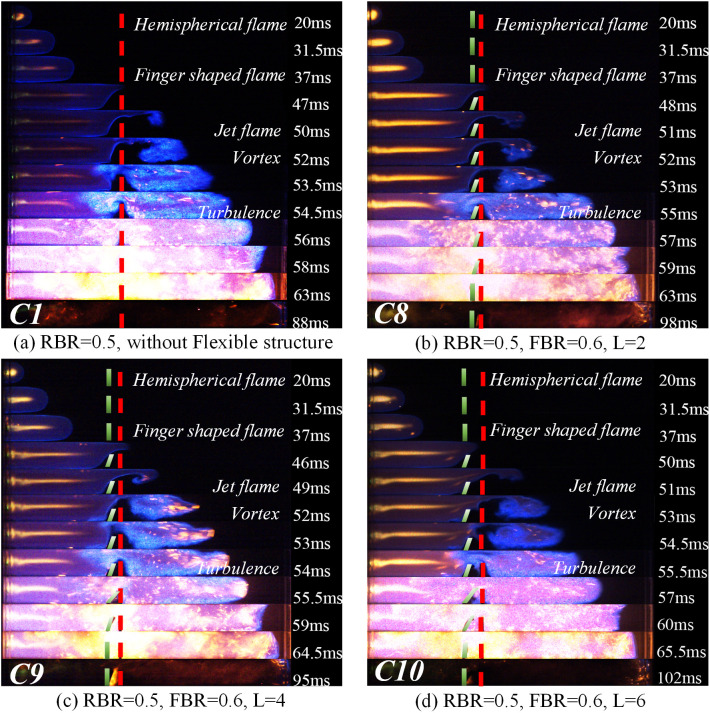
Flame Propagation Law at FBR = 0.6.

Changes in the flame’s structure and velocity are mainly influenced by the interaction between the flame and the unburned gas in front of the flame. In the explosion environment where structures exist, the change of the structure and velocity of the flame front is the focus of understanding the flame propagation law affected by structures. In this paper, images of the various stages of the flame propagation process are collected by a high-speed camera system, and the influence of the coexistence of rigid and flexible structures on the flame structure is obtained under different working conditions. Fig 4 divides the captured flame process into five stages: initial flame stage, flame deformation stage, turbulence development stage, explosion intensification stage, and late stage of flame. The right side of the flame figures corresponds to the characteristics of each stage. Fig 5 illustrates the change in the flame structure when the blockage rate of the purely rigid structure is 0.5 (RBR = 0.5) and the flexible structure has a blockage rate of 0.4 (FBR = 0.4) and the flexible structure is placed in front of the rigid structure. Fig 6 represents the change in the flame structure when the blockage rate of the pure rigid structure is 0.5 (RBR = 0.5) and when the blockage rate of the flexible structure is 0.5 (FBR = 0.5) and the flexible structure is placed in front of the rigid structure. Fig 7 represents the change in the flame structure when the blockage rate of the pure rigid structure is 0.5 (RBR = 0.5) and the blockage rate of the flexible structure is 0.6 (FBR = 0.6) and the flexible structure is placed in front of the rigid structure. The flame images show that the explosion flame mainly goes through four stages: hemispherical, finger, planar, and tulip [19].

In the flame graphs of Figs 5 and 7, it can be observed that the flame takes on a hemispherical shape about 20 ms after ignition. This is because the homogeneously mixed quiescent explosive gases start to expand, and the products of combustion bring about volumetric expansion, thereby driving the flame speed [20].

After the flame structure experiences a spherical shape, it accelerates in the finger-shaped stage. From Figs 5 and 7, it can be observed that the flame starts to deform around 37ms under the influence of the rigid/flexible structure. The combustion channel narrows under the action of the rigid/flexible structure. The flame is squeezed and starts to narrow above the rigid/flexible structure, which encourages the flame to pass through the rigid/flexible structure more quickly and spreads combustion to the unburned area behind the structure. Based on the flame acceleration model proposed by Wingerden and Zeeuwen [21]:


$$\begin{array}{c} V_{f}=\sigma \cdot S_{L}\cdot \frac{A_{l}}{A_{o}} \end{array}$$ (2)

The increase in flame front velocity during flame propagation is mainly due to the increase in flame area. The flame forms a shear layer above the rigid-flexible structure and a reflux zone behind it because the rigid-flexible structure obstructs the channel. The surface area of the flame increases as it passes through the rigid-flexible structure because of expansion and fluid instability, increasing flame front velocity [22]. The shear layer falling above the rigid-flexible structure and the Kelvin-Helmholtz instability of the fluid form turbulence behind the rigid-flexible structure [23]. The flame front is sucked into the reflux zone, the laminar flow behind the structure transforms to turbulence, and the flame area rises rapidly, accelerating the flame combustion and explosion.

By comparing Figs 5 and 7, it can be seen that a vortex with wider coverage is generated above the pure rigid structure simultaneously. However, the flame propagation process is affected by the characteristics of the flexible structure when the flexible structure is in front of it. The magnitude of the vortex in the rear of the rigid-flexible structure has a close relationship with the blockage rate and spacing of the flexible structure. By observing the flame propagation image, it can be found that around 52ms after ignition, the pure rigid structure forms a larger vortex than the other cases, which is because in the case of the flexible structure front, when the flame is in contact with the flexible structure, because of its characteristics, the flexible structure is accelerated and tilted backward by the flame, and deformation occurs. The deformation of the flexible structure reduces the true blockage rate of the structure and, at the same time, widens the flame channel, making the flame take longer to reach the same position compared to a purely rigid structure.

Flame propagation characteristics are affected by the blockage rate and the spacing between the rigid and flexible structures. Flame propagation in an obstructed channel creates pockets of fresh fuel mixture between the rigid and flexible structures [24]. Due to delayed combustion in the pockets, the combustion gases produce a powerful jet of gas in the unobstructed part of the channel. The jet causes the flame tip to propagate faster, which creates new pockets that create positive feedback between the flame and the jet gas, thus accelerating the flame, a phenomenon known as the “pocket effect” [24]. In this experiment, the flame propagates at a very high speed in the unobstructed portion of the channel, leaving regions of unburned mixture between the rigid and flexible structures, which would burn later.

From Figs 5 and 7(b)–7(d) observed that, with the increase of the spacing between the rigid and flexible structures, the larger the “pockets” between the rigid and flexible structures, and thus the more unburned mixture stored between the “pockets”, when the flame tip arrives at the top of the flexible structure, the flame is swirled into combustion within the “pockets” composed of rigid and flexible structures, and the unburned mixture burns rapidly within the “pockets”, generating expanding gases and pushing the flame to accelerate.

### Flame front velocity

The flame front velocity V in the pipe depends on the flame propagation distance difference ΔL and the corresponding time difference Δt. The flame propagation distance difference ΔL is determined by the position of the flame front $\left(L_{\text{1}},L_{\text{2}}\right)$ corresponding to the two time points $\left(t_{\text{1}},t_{\text{2}}\right)$. The formula for calculating the flame front velocity is shown below:


$$\begin{array}{c} V=\frac{\Delta L}{\Delta t}=\frac{L_{\text{2}}-L_{\text{1}}}{t_{\text{2}}-t_{\text{1}}} \end{array}$$ (3)

**Fig 8:**
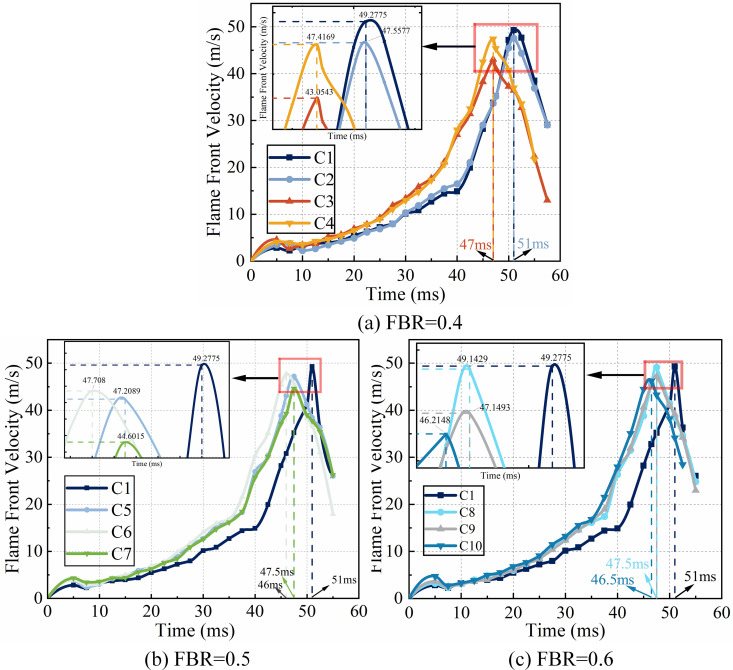
Flame front velocity under different FBR conditions.

The flame front velocity can be calculated by eq. (3), and the obtained data are plotted on a line graph to obtain Fig 8. From Fig 8, it can be found that regardless of the blockage rate of the flexible structure and the rigid-flexible spacing, the rigid structure has a higher flame speed compared to the flexible structure front, which is 49.2775 m/s. From Fig 8(a), it can be seen that, when the blockage rate of the rigid structure is 0.5 and the blockage rate of the flexible structure is 0.4, the flame speed of the flexible structure front is significantly smaller than that of the rigid structure, of which the flexible structure front is 4 cm, the flame speed is the lowest, which is 43.0543 m/s. From Fig 8(b), it can be seen that when the blockage rate of the rigid structure is 0.5 and the blockage rate of the flexible structure is 0.5, the flame velocity of the flexible structure front case are significantly smaller than that of the purely rigid structure, in which the flame velocity is the lowest when the flexible structure front is 6 cm, which is 44.6015 m/s. Moreover, it is clearly found in the Fig 8(b), that the flame peak arrives at an earlier time in the flexible front case. From Fig 8(c), it can be seen that when the blockage rate of rigid structure is 0.5 and the blockage rate of flexible structure is 0.6, except for the flame velocity of flexible front 2 cm which is closest to that of pure rigid structure, the flame velocity in the case of flexible front is significantly smaller than that of pure rigid structure, in which the flame velocity is the lowest in the case of flexible structure front 6 cm, which is 46.2148m/s. In the initial few milliseconds after ignition, the flame appears as a spherical flame. The initial increase in its propagation speed primarily stems from the rapid expansion of the flame front and the hydrodynamic instability caused by thermal expansion, which temporarily outweighs other suppression effects. Subsequently, the flame front rapidly expands to the side walls of the pipeline, and the propagation mode transitions from spherical propagation to confined finger-like propagation. This transition leads to two main consequences: firstly, the quenching effect on the pipeline wall surface is significantly enhanced, resulting in substantial heat and active radical losses; secondly, the flow of unburned gas in front of the flame is restricted, generating a reverse pressure. These two effects work together to cause a temporary increase in flame speed in the initial stage, followed by a subsequent decrease. The flame starts from the ignition end and touches the rigid-flexible structure at about 40ms. It can be seen that the flame front velocity function curve tends to be flat and rises slowly before 40ms. After passing through the rigid-flexible structure, the velocity function curve suddenly becomes steeper, rises faster, and declines slowly after reaching the peak. The main reason for this phenomenon is that the rigid-flexible structure makes the cross-section in the pipe smaller, resulting in the unburned gas body flow velocity increases in the narrow place, and the flame front velocity starts to surge when the flame passes through the rigid-flexible structure. The rise in initial flame front velocity leads to a rise in vortex extent, which promotes the transition of the unburned gas from laminar to turbulent flow, leading to the appearance of peak flame velocity.

The comparison of flame velocities shows that the rigid structure allows the flame to gain more velocity. The flexible structure, on the other hand, attenuates the velocity by softening most of the forward shock wave and disturbing the flame through self-generated deformation.

**Fig 9:**
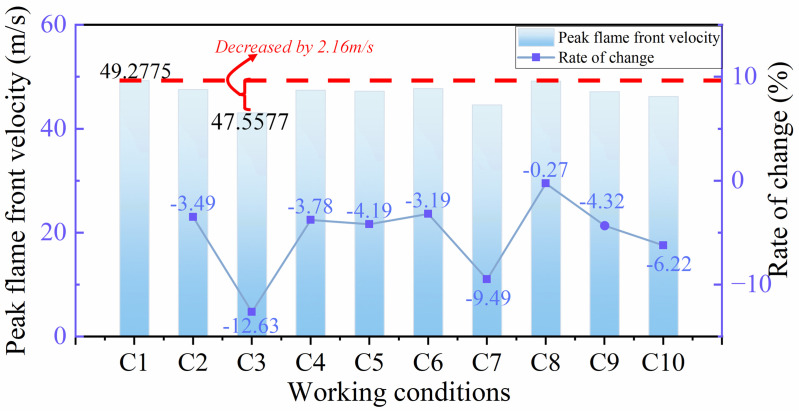
Peak value and change rate of flame front velocity under different working conditions.

Fig 9 shows the peak flame front velocity and the rate of change of peak velocity for various working conditions. From Fig 9, it can be seen that the flame front velocity is lower than the pure rigid structure condition in all flexible structure front conditions. Among them, the most obvious decrease in flame velocity is 2.16 m/s when the blockage rate of flexible structure is 0.4 and the rigid-flexible spacing is 4 cm; the smallest decrease in flame velocity is only 0.13 m/s when the blockage rate of flexible structure is 0.6 and the rigid-flexible spacing is 2 cm. From the rate of change of the velocity of the individual conditions for the pure rigid structure, the rate of change is less than 0, which also indicates that the flame front velocity of the flexible This also indicates that the flame front speed of the structure front condition is smaller than the pure rigid structure flame speed. Among them, when the blockage rate of the flexible structure is 0.4 and the rigid-flexible spacing is 4 cm, the flame speed change is the most obvious, and the change rate is −12.63%; when the blockage rate of the flexible structure is 0.6 and the rigid-flexible spacing is 2 cm, the flame speed change is the smallest, and the change rate is −0.27%. Fig 9 shows the flexible structure front can significantly reduce the flame front velocity under most working conditions. According to Damir [24], flames form “pockets” of fresh fuel mixture between the rigid and the flexible, so in general, the smaller the interval between the rigid and the flexible, the less unburned gas mixture in the “pockets” and the more effective it is for the attenuation rate of the flame peak. Fig 9 shows that with the rigid-flexible interval of 2 cm when the C2 and C5 conditions, the flame front velocity attenuation effect is more obvious. However, the actual situation is not absolute, but also need to pay attention to the height ratio of rigid-flexible structure and rigid-flexible structure spacing. As shown in Fig 9, the working condition with the best attenuation effect of front velocity is C3; that is, the flexible structure with a blockage rate of 0.4 is placed 4 cm away from the rigid structure, which is the joint effect of the height ratio of the rigid and flexible structures and the spacing between them. Because the flexible structure is lower than the rigid structure, the flame passes through the rigid and flexible structure to form a kind of gradient upward trend, which greatly slows down the flame and the flexible structure of the front impact. In addition, the gap between the rigid and flexible structures is small, and there is less unburned mixture, so the flame front velocity is attenuated to a certain extent. In the C8 condition, the flexible structure blockage rate of 0.6, greater than the rigid structure blockage rate of 0.5, coupled with the rigid and flexible structure spacing is small at this time, the rigid structure in the flexible structure deformation in the back of the support role, reducing the degree of deformation of the flexible structure, thus escalating the explosion.

### Explosion overpressure

**Fig 10:**
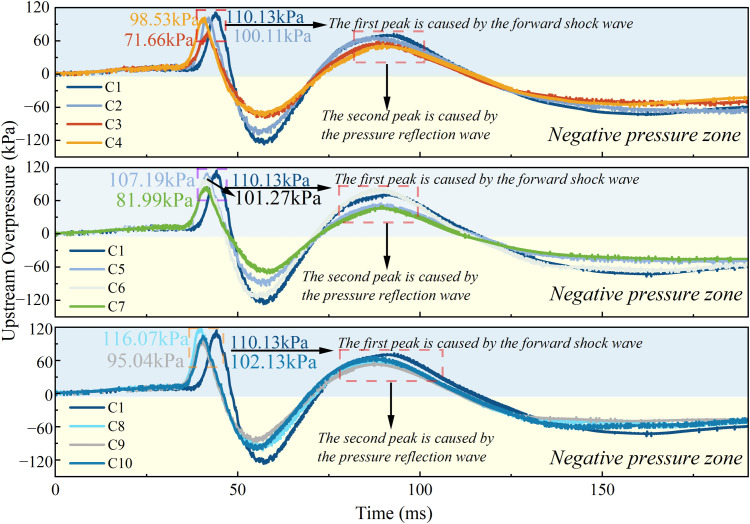
Influence of blockage rate of flexible structure and space on overpressure upstream of explosion.

Fig 10 shows the upstream overpressure of the flame for each working condition. From the three figures, it is obvious that that the upstream pressure of the purely rigid structure is greater than the vast majority of the flexible front conditions. When the flexible structure blockage rate is 0.4, the upstream pressure at the flexible structure front is significantly lower than the upstream pressure at the pure rigidity. Among them, the upstream pressure is the smallest when the blockage rate of flexible structure is 0.4 and the rigid-flexible spacing is 4 cm, which is 71.66 kPa. When the blockage rate of flexible structure is 0.5, the difference between the upstream pressure at the time of flexible structure front and the upstream pressure at the time of purely rigid structure is smaller. Among them, when the blockage rate of flexible structure is 0.5 and the rigid-flexible spacing is 6 cm, the upstream pressure is the smallest, 81.99 kPa. When the blockage rate of flexible structure is 0.6 and the rigid-flexible spacing is 2 cm, the flexible structure front plays a reverse excitation effect on the upstream pressure, and at this time, the upstream pressure is larger than that of the pure rigid structure, which is 116.07 kPa.

From Fig 10, it can be observed that the upstream pressure after ignition shows a slow rising trend until the flame shock wave began to contact the first pressure sensor, the flame explosion overpressure begins to rise sharply, and at this time reaches the first overpressure peak; shock wave sweeps through the first pressure sensor, the negative pressure zone then appears; with the filling of the late unburned mixture, the pressure quickly recovers and rises sharply. With the acceleration of the flame speed, the flame structure changes, and the role of turbulence makes the pressure peak. After the pressure peaks, it decays exponentially, forming a brief negative pressure zone and gradually returning to ambient pressure, and the pressure waveform shows the classical Friedlander explosion waveform [25].

**Fig 11:**
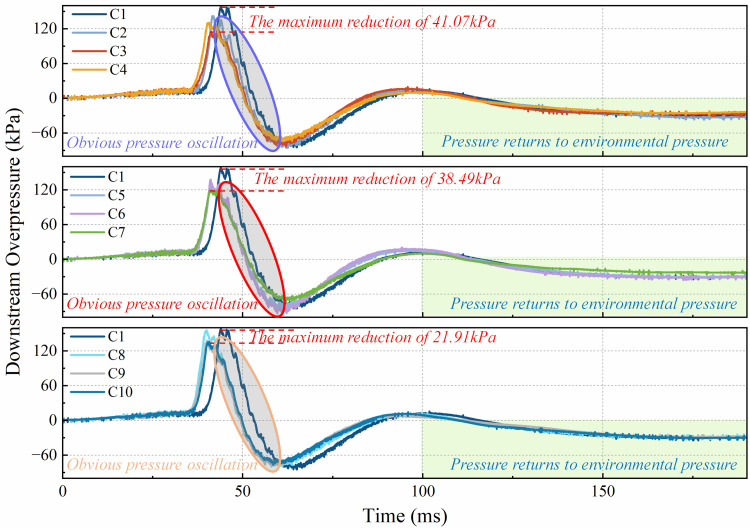
Influence of blockage rate of flexible structure and space on overpressure downstream of explosion.

Fig 11 shows the downstream overpressure of the flame for each working condition. From the three figures, it is obvious that the downstream pressure of the pure rigid structure is greater than that of all flexible front conditions. When the flexible structure blockage rate is 0.4, the downstream pressure at the flexible structure front is significantly lower than the downstream pressure at the pure rigidity. Among them, the downstream pressure decreases most obviously when the blockage rate of flexible structure is 0.4 and the rigid-flexible spacing is 4 cm, which decreases 41.07 kPa. When the blockage rate of flexible structure is 0.5, the downstream pressure in the case of flexible structure front is significantly lower than that in the case of pure rigidity. When the blockage rate of the flexible structure is 0.5 and the rigid-flexible spacing is 6 cm, the downstream pressure decreases most obviously, decreasing by 38.49 kPa. When the blockage rate of the flexible structure is 0.6 and the rigid-flexible spacing is 2 cm, the downstream pressure attenuation effect of the flexible structure front on the downstream pressure is weak. When the flexible structure blockage rate was 0.6 and the rigid-flexible spacing was 6 cm, the downstream pressure decreased most significantly, by 21.91 kPa.

Fig 11 shows the explosion overpressure graph downstream of the flame for each operating condition. Similar to the upstream, the downstream overpressure peak is caused by the flame skimming over the second pressure sensor, with an exponential decrease after the peak and a brief negative pressure region followed by a return to ambient pressure, showing the classic Friedlander explosion waveform. However, unlike the upstream overpressure, it is clear that the downstream overpressure peaks are accompanied by fluctuations due to repeated vibrations of the pressure curve, a phenomenon known as the “multi-peak phenomenon”. This phenomenon is mainly due to the membrane rupture during the propagation of the explosion shock wave, resulting in a sharp drop in the overpressure generated by the explosion. After the pressure release, due to the increase of vortices, the explosion overpressure is characterized by oscillations with increasing turbulence intensity.

Through the observation of Figs 10 and 11, it can be noted that the flexible structure front case, the flame explosion overpressure in most cases, has obvious attenuation. Especially under working condition C3, the overpressure of the flame explosion is the most significant. This is because the flame shock wave rises along the gradient built by the rigid and flexible structure, greatly reducing the frontal impact of the flame on the flexible structure. In addition, the spacing between the rigid and flexible structures is small, and there are fewer unburned mixtures, so the overpressure of flame explosion is attenuated to a certain extent. In contrast, the C8 condition has a poor attenuation effect on flame explosion overpressure and even promotes explosion overpressure. The main reason for this phenomenon is that the blockage rate of the flexible structure (RBR = 0.6) is too high. Excessive flexible blockage rate coupled with a small rigid-flexible interval at this time, in the flexible structure to receive the flame shock wave impact of the moment, the rigid structure plays a supporting role on the flexible structure, so that the degree of deformation of the flexible structure becomes smaller, so as not to achieve the desired reduction in the explosion overpressure effect. To sum up, the flexible structure front can reduce the flame explosion overpressure in the pipeline, which is consistent with the previous conclusion.

**Fig 12:**
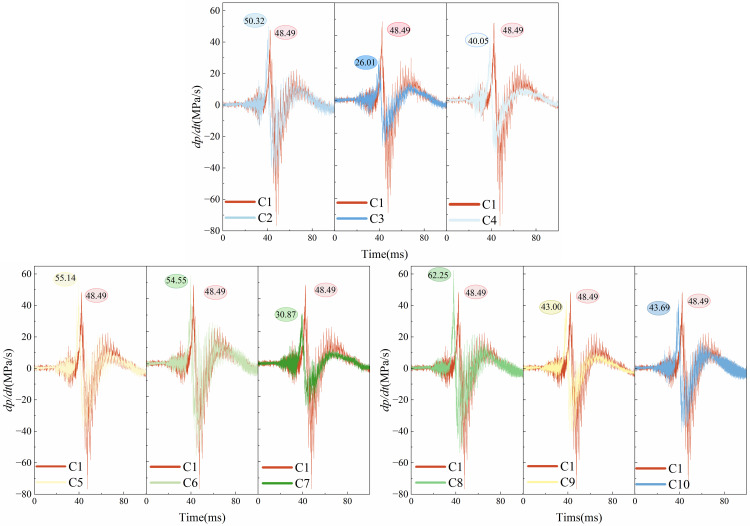
Upstream pressure rise rate figure.

Fig 12 shows the upstream pressure rate of change $\frac{dp}{dt}$. The rate of pressure change visualizes how drastically the pressure changes over time at a given moment. As shown in figures, the upstream pressure change trend for each condition is shown in figures. The change first exhibits a slow rise, and then the pressure rate rises to reach f the peak explosion overpressure. After the peak, the pressure gradually decreases, the rate of change of pressure also gradually declines, the pressure fluctuation is getting smaller and smaller. The overall trend is first up and then down and then back to 0. The higher the value of the rate of change of pressure means that a short period of time to release more energy, i.e., the explosion intensity, destructive power. In the figures, it can be seen that the maximum pressure change rate occurs in the flexible structure blockage rate of 0.6, the flexible front 2 cm, 62.25MPa/s. At this time, the blockage rate of flexible structure is higher than the blockage rate of rigid structure, which occupies more flame paths in the pipeline, thus prompting the flame from the rigid and flexible structure of the narrow place above the passage. At the same time, the positive impact of the flame leads to the deformation of the flexible structure, but at this time, the rigid-flexible spacing is 2 cm, after a small deformation of the flexible structure, the rigid structure plays a supportive role after it, reducing the deformation amplitude, which results in the flame can pass through the channel is still limited, and ultimately leads to a sudden increase in the upstream pressure of the flame, the rate of change of pressure is large, and the flame is changing dramatically in this period of time. At the same time, it can be found in the figures, the smallest pressure change rate appears in the flexible structure blockage rate of 0.4, flexible front 4 cm, for 26.01MPa/s. At this time, the blockage rate of flexible structure is lower than the blockage rate of rigid structure, in the flame directly in front of the formation of a ladder, the flame front to follow the trend of the flame, reducing the flame of the forward shock. At the same time, the deformation of the flexible structure itself for the flame front to provide a wider flame channel, coupled with the rigid-flexible spacing of 4 cm, not only for the flexible structure to provide sufficient deformation space, but also reduces the rigid-flexible spacing in the middle of the storage of the unburned gas, which ultimately leads to the upstream of the flame pressure rises smaller, the pressure change rate is small, the flame in this period of time, the smaller the degree of intensity of the explosion.

**Fig 13:**
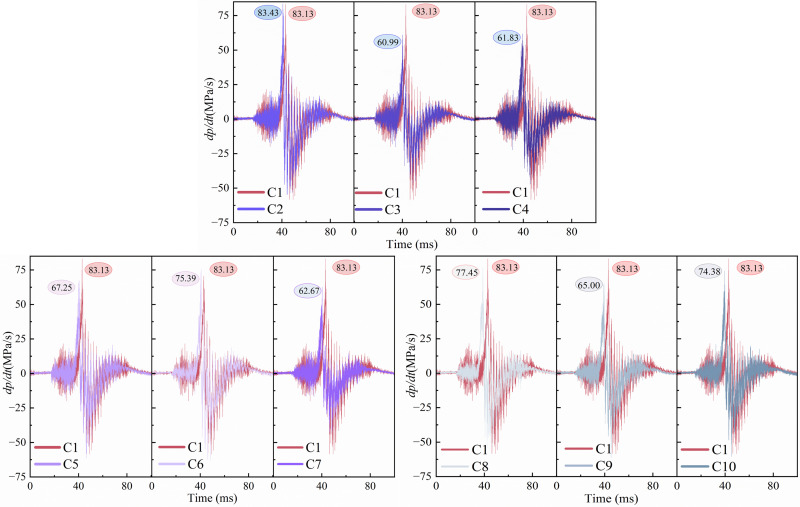
Downstream pressure rise rate figure.

Fig 13 shows the rate of change of downstream pressure $\frac{dp}{dt}$. The flame front releases energy in the middle and lower reaches after passing through the rigid-flexible structure, causing the downstream pressure to rise sharply over the upstream. From the figures, it can be found that the downstream pressure change rate is generally higher than the upstream pressure change rate. Among them, the largest downstream pressure change rate appears in the flexible structure blockage rate of 0.4, flexible front 2 cm, for 83.43MPa/s. At this time, due to the influence of the upstream flexible structure blockage rate and rigid-flexible spacing, the explosion overpressure is large, after passing through the rigid-flexible structure, the flame in the downstream of the concentration of the release of energy, which leads to the flame pressure changes dramatically. The smallest downstream pressure change rate occurs in the flexible structure blockage rate of 0.4, flexible front 4 cm, 60.99MPa/s. At this time, due to the upstream explosion overpressure is large, the explosion reaction is intense, so the release of energy in the middle and lower reaches of the energy is weaker, resulting in a smaller downstream pressure change rate.

**Fig 14:**
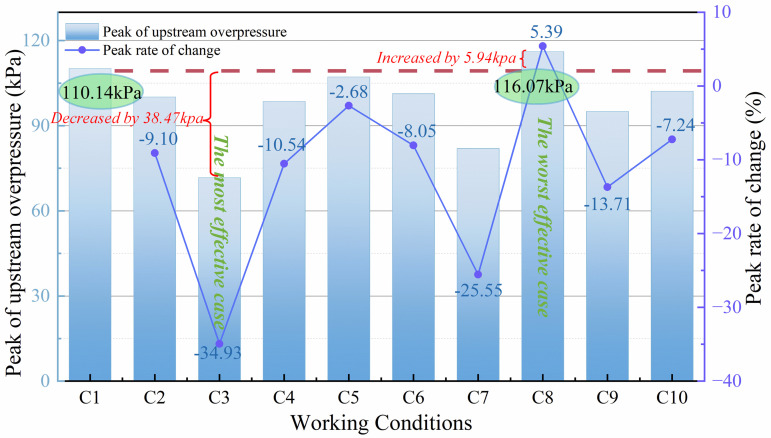
Peak value and change rate of upstream pressure.

Fig 14 shows the upstream peak explosion overpressure and the rate of change of the peak pressure for various working conditions. From Fig 14, it can be seen that the peak explosion overpressure is significantly decreased for all the working conditions except for C8. Among them, the explosion overpressure decreases most in C3, when the blockage rate of the flexible structure is 0.4 and the rigid-flexible spacing is 4 cm, it decreases by 38.47 kPa. when the blockage rate of the flexible structure is 0.6 and the rigid-flexible spacing is 2 cm, the high blockage rate of the flexible structure as well as the narrow spacing of the rigid-flexible structure play an incentive role in the explosion overpressure, which makes the explosion overpressure increase instead of decreasing, and increases by 5.94 kPa. from the various conditions for the upstream pressure peak change rate of the pure rigid structure, except for C8, the change rate is less than 0, which indicates that except for C8, the upstream pressure peak is significantly attenuated. Among them, the most decrease is in C3, with a change rate of −34.93%, and C8 increases instead of decreasing, with a change rate of 5.39%.

**Fig 15:**
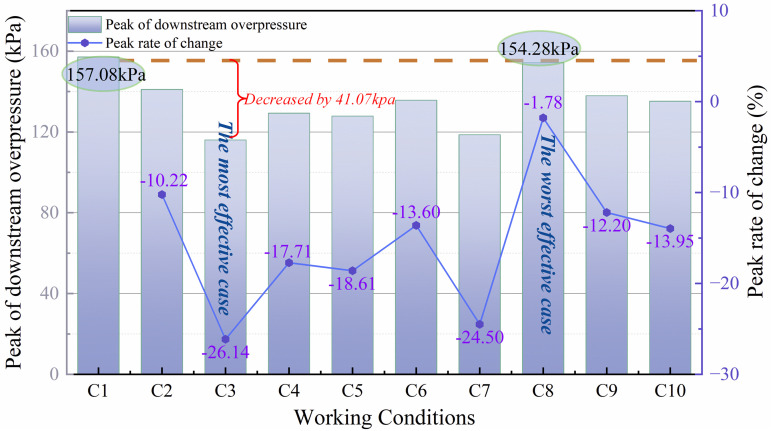
Peak value and change rate of downstream pressure.

Fig 15 shows the downstream peak explosion overpressure and the rate of change of the peak pressure for various operating conditions. From Fig 15, it can be seen that the peak explosion overpressure is decreased for all conditions compared to the downstream overpressure of the purely rigid structure. Among them, the explosion overpressure decreases the most is C3, i.e., when the blockage rate of flexible structure is 0.4 and the rigid-flexible spacing is 4 cm, it decreases by 41.07 kPa. when the blockage rate of flexible structure is 0.6 and the rigid-flexible spacing is 2 cm, the high blockage rate of the flexible structure as well as the narrow spacing of rigid-flexible structure has limited attenuation of explosion overpressure, which decreases only by 2.8 kPa. From the rate of change of downstream pressure peak for pure rigid structure for each working condition, the rate of change is less than 0 for all conditions, and the downstream pressure peak is significantly attenuated for all conditions. Among them, the most decrease is C3 with a decrease rate of −26.14%, and the least decrease is C8 with a decrease rate of −1.78%.

### Mechanism analysis

Flame propagation is when the chemical reaction between a combustible gas and an oxidizer releases energy and drives the unburned mixture forward. Upon ignition of a methane/air-premixed gas, the expansion of the combustion products drives the flame to propagate forward [26–27]. In the initial stage of flame propagation, the flame shape is usually hemispherical, and the flame velocity is low, which is mainly influenced by the expansion of the combustion products. As the flame propagation distance increases, the flame gradually accelerates, and the shape evolves from hemispherical to finger-shaped, planar flames, and eventually tulip-shaped flames. The acceleration of the flame speed in this process is mainly due to the propulsive effect of the expansion of the combustion products on the unburned mixture.

**Fig 16:**
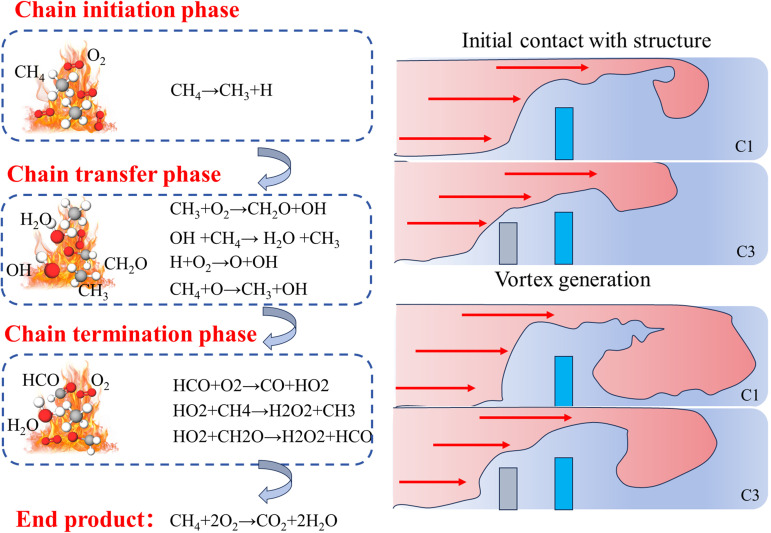
Mechanism analysis figure.

The flow field changes significantly when the flame comes into contact with the rigid-flexible structure. The rigid-flexible structure “stretches and folds” the flame surface, increasing the surface area of the flame and leading to an increase in the heat release rate. The flexible structure, through its deformation, slows down the “stretching and folding” effect of the flame and reduces the combustion rate. Compared with the rigid structure, when the flexible structure is in front, the vortex formed in the flame propagation process is smaller in scope, which reduces the center pressure of the pipe and weakens the flame vortex, thus reducing the flame speed. As shown in the upper right of Fig 16, at the initial stage of flame contact with the rigid-flexible structure, the purely rigid structure, because of its blockage rate remains constant, makes the flame pass through the channel narrower, thus accelerating the flame front velocity, so that the vortex propagation in the rear of the rigid structure is farther away and wider in scope. In the case of the flexible structure front, especially when the flexible structure is slightly lower than the rigid structure, the gradient formed by the rigid-flexible structure makes the flame shock wave rise step by step, which greatly reduces the intensity of the flame shock wave, and also reduces the propagation distance and range of the vortex behind the rigid-flexible structure.

Explosion pressure wave is another important phenomenon in the explosion process, and its formation is closely related to flame propagation. When the flame propagation speed reaches a certain level, the rapid expansion of combustion products will form a pressure wave [28–30]. The propagation velocity of pressure wave is usually much higher than that of flame, and it interacts with flame in the process of propagation. The peak pressure, waveform and attenuation law of pressure wave are important indicators to measure the destructive force of explosion.

The propagation of pressure waves is affected by several factors, including rigid and flexible structures, wall reflections, and turbulence [31]. When a pressure wave reaches a rough wall, wall reflections cause the flow to spread rapidly in the combustion chamber, and turbulent kinetic energy is transferred to the initially stationary mixture. This reflection enhances the intensity of the pressure wave and may lead to multiple peaks in the pressure wave (i.e., the “multi-peak phenomenon”). The multi-peak phenomenon is mainly due to repeated oscillations of the pressure wave due to reflections from rigid and flexible structures or wall surfaces encountered during the propagation of the pressure wave [32]. The pressure wave propagation is significantly affected when the flexible structure is fronted. The flexible structure absorbs part of the pressure wave energy through deformation, reducing the peak pressure of the pressure wave. In addition, the flexible structure’s deformation reduces the pressure wave’s direct impact on the rigid structure, thus reducing the destructive force of the explosion.

The rigid-flexible structure is one of the key factors affecting flame explosion propagation. Flexible structure blockage rate, rigid and flexible structure spacing on the flame propagation speed and the formation of the explosion pressure wave have a significant impact. Different blockage rates of flexible structures affect the flame propagation speed and explosion pressure differently. Flexible structures with high blockage rates significantly accelerate flame propagation and increase explosion pressure. Purely rigid structures accelerate flame propagation by blocking the flame propagation path and enhancing the turbulence effect of the flame. Flexible structures, on the other hand, slow down flame propagation through deformation and reduce explosion pressure.

**Fig 17:**
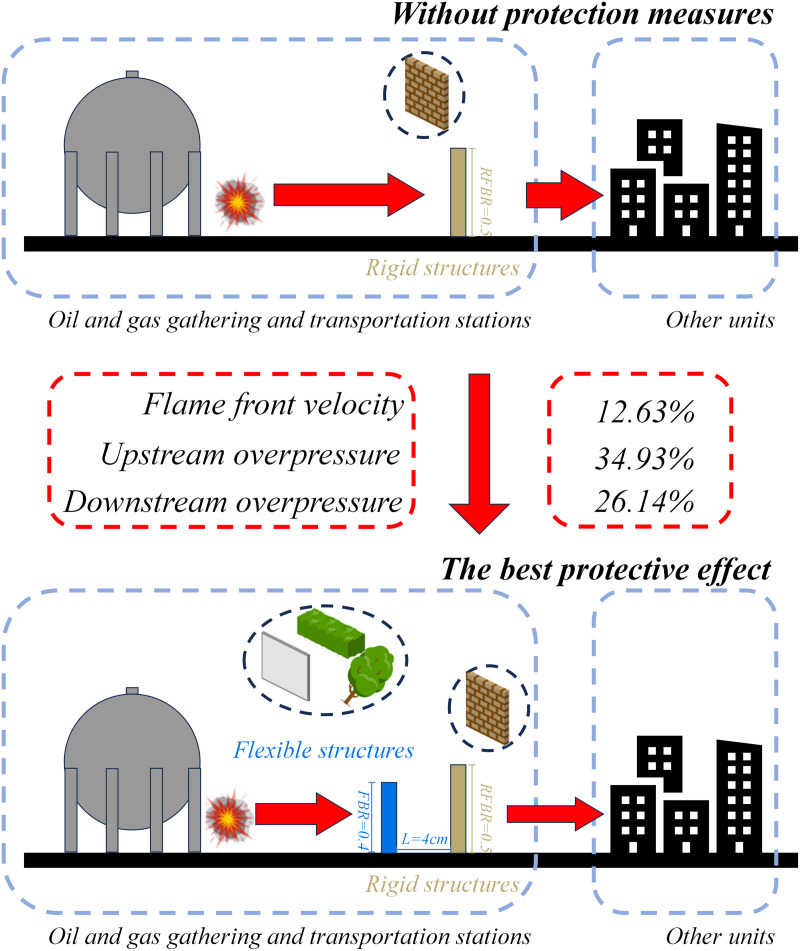
Schematic diagram of safety protection for flexible structures in oil and gas gathering and transportation station.

Fig 17 shows the safety protection plan applicable to oil and gas gathering and transportation stations based on the research results of this article. As shown in Fig 17, without protective measures, the explosion intensity is enhanced through the inherent rigid structure, causing casualties and property damage to adjacent units. On the premise of flexible structure protection, according to the research results in this paper, the best protection effect is C3, that is, the blockage rate of flexible structure is 0.4, and the rigid flexible spacing is 4 cm. Therefore, from the blockage rate of the three flexible structures studied in this paper, the flexible structure should be set in the oil and gas gathering station, and the blockage rate of the flexible structure should be lower than the rigid structure, and the rigid flexible structure spacing is equal to the height of the flexible structure. The protection plan designed based on this situation can effectively reduce the speed, overpressure, and intensity of the explosion flame.

## Conclusion

This paper mainly uses the method of combining experimental research and theoretical analysis to study and analyze the methane-air premixed flame propagation phenomena and laws, including the dynamic change characteristics of the flame front surface, flame front velocity and the explosion flame pressure characteristics for the existence of a variety of structures in the Oil and gas gathering and transportation station environment. Based on the explosion protection requirements of oil and gas gathering and transportation stations, this experiment analyzes the impact of the blockage rate of flexible structures and the spacing between rigid and flexible structures on explosion flames. It identifies the layout of rigid and flexible structures that can better suppress flames, providing a practical reference for reducing the hazards of Oil and gas explosions and enhancing on-site safety.

(1) When the flexible structure is placed in front of the rigid structure, the deformation characteristics of the flexible structure can disturb the flame vortex, increase the probability of collision between free radicals of combustible gases and the flexible structure, so as to weaken the strength of the flame shock wave and achieve the purpose of protecting the rigid structure.
(2) Under most working conditions, placing the flexible structure in front can attenuate the peak velocity of the flame front and the overpressure of flame explosion. The main influencing factors are the blockage rate of flexible structures and the spacing between rigid and flexible structures. Generally, the flame front’s peak velocity and the flame explosion’s overpressure are positively correlated with the blockage rate of flexible structures. When the blockage rate of flexible structure is lower than or equal to the blockage rate of rigid structure, the experimental results conform to the flame front velocity and flame explosion overpressure increase with the expansion of rigid-flexible structure spacing, especially in the working condition C3 (FBR = 0.4 < RBR = 0.5, L = 4), the explosion protection effect is the most obvious.
(3) However, the actual situation is not to separate the blockage rate of flexible structures and the spacing between rigid and flexible structures. These two factors should be linked together, and they affect the characteristics of flame explosion propagation together. When FBR > RBR, the smaller the rigid and flexible structure spacing is, the flame explosion will be promoted.

## Supporting information

S1 File The velocity of flame. (XLSX)

S2 File The overpressure and the change rate of flame. (XLSX)
